# Remembering to whom we transmit information during pandemics: the effect of face masks on destination memory

**DOI:** 10.1007/s10339-023-01126-4

**Published:** 2023-02-08

**Authors:** Raquel Pinto, Diogo Lima, Beatriz Mello, Pedro B. Albuquerque

**Affiliations:** School of Psychology, University of Minho, Campus de Gualtar, 4710-057 Braga, Portugal

**Keywords:** COVID-19, Surgical masks, Destination memory, Facial recognition

## Abstract

Considering the global pandemic we currently experience, face masks have become standard in our daily routine. Even though surgical masks are established as a safety measure against the dissemination of COVID-19, previous research showed that their wearing compromises face recognition. Consequently, the capacity to remember to whom we transmit information—destination memory—could also be compromised. In our study, through a between-participants design (experiment 1) and a within-participants design (experiment 2), undergraduate students have to transmit Portuguese proverbs to masked and unmasked celebrity faces. Following our hypothesis, participants who shared information with masked faces had worse destination memory performance than those who shared information with unmasked faces. Also, we observed lower recognition for masked faces compared to unmasked faces. These results were expected since using a surgical mask affects facial recognition, thus making it harder to recognize a person to whom information was previously transmitted. More importantly, these results also support the idea that variables associated with the recipient’s face are important for destination memory performance.

## Introduction

In 2020, a global pandemic affected the world and led many countries to close their borders and confine their population to their households. Even though the world is returning to normality, with the population regaining most of their free circulation and widespread vaccination, the consequences of COVID-19 are still present in our everyday lives. For example, the use of face masks became standard in our daily routine as a result of the adoption by most countries of policies that aim to avoid the spread of the pandemic, particularly in public places, as recommended by the World Health Organization (2020).

Although the importance of masks in effectively reducing virus transmission has been demonstrated (Howard et al. 2021), their use may lead to difficulties in facial processing. When using a face mask, about 60 to 70% of a person’s face is covered, hiding parts of it that are essential, for example, for its identification (e.g., Tsao and Livingstone 2008), for judging friendliness or attractiveness, and for emotion detection (Goldstein and Brockmole 2017).

Face recognition has been linked to holistic face processing (Freud et al. 2020), i.e., faces are perceived as a whole rather than a combination of different components (Farah et al. 1998; Maurer et al. 2002), with holistic processing being interrupted by the presence of facial occlusions, for example, the use of sunglasses, hats, or scarves that partially cover the face. Even in the absence of occluding objects, shadows, or intense light caused by a flash in a picture may harm holistic face processing. Religion is a very particular context in which it is not uncommon to encounter facial occlusions through the use of niqabs, a garment that covers the face, used by Muslim women that leave the eye region uncovered (Wang et al. 2015).

However, it is also important to note different contributions of the upper and lower facial features for emotion identification and face recognition (Eisenbarth and Alpers 2011; Sadr et al. 2003). For example, in a study that applied eye-tracking methodology, it was found that people spent more time looking (i.e., longer fixations) at the eyes to detect sad facial expressions and at the mouth for happiness (Eisenbarth and Alpers 2011). Also, emotions whose discrimination depends heavily on mouth configuration, such as sadness, happiness, and anger, were often misinterpreted as neutral when expressed covered by a mask (Carbon 2020). Regarding face recognition, removing the eyebrows from familiar faces significantly impaired recognition accuracy compared to eliminating the eyes (Sadr et al. 2003), suggesting that different features contribute differently to face recognition. When comparing configural facial features (e.g., eye distance and face proportion) and face parts (e.g., lip thickness and eye shape), Abudarham et al. (2019) observed that configural facial features were less important for face identification, thus reinforcing the importance of crucial facial parts for the identification of faces. Also, regarding facial expressions (see Calder et al. 2000), it was observed that a person relies on changing information of local facial regions to understand what emotion is expressed or changed in real-time (e.g., Tobin et al. 2016). So, one of the most significant current challenges is determining which facial characteristics are more important in identifying faces or emotions (Sadr et al. 2003).

However, although different features appear to contribute differently to face recognition (Carbon 2020; Sadr et al. 2003), more recently, and already during the pandemic context brought by COVID-19, all evidence showed that the use of face masks affects face processing (Carragher and Hancock 2020; Freud et al. 2020). Using a surgical mask disrupts face processing, affecting facial and emotional identification and hindering memory for faces. It has also been found that using a face mask affects both perceived attractiveness (Patel et al. 2020) and face recognition (e.g., Freud et al. 2020).

Regarding face recognition, Carragher and Hancock (2020) sought to understand how a face mask can affect face processing by using a face-matching procedure (i.e., if two presented photographs showed the same person's face). The authors compared three different conditions: both faces without a mask (control), one of the faces wearing a mask and the other displayed without one (mixed), and both faces wearing a mask (masked). Results revealed better face-matching performance in the control condition, where both faces were presented without a mask, suggesting that using a surgical mask, even if only in one of two stimuli, leads to an impairment in face perception and, ultimately, to difficulties in face recognition (Carragher and Hancock 2020).

Even though Carragher and Hancock’s (2020) results suggest that using a surgical mask impairs facial recognition, it is important to note that the task used in this study was a face-matching task, which differs considerably from a recognition memory test. To understand whether a face mask impairs facial recognition, Freud et al. (2020) instructed participants to complete the Cambridge face memory test (Duchaine and Nakayama 2006). Their study had two conditions: one in which the faces were presented with a face mask and one in which the presented faces were unmasked. Indeed, results showed that wearing a face mask hindered face recognition.

Bearing in mind that previous literature (Carragher and Hancock 2020; Freud et al. 2020; Sadr et al. 2003) suggests that face recognition is worsened by surgical mask use, it is essential to understand how our interpersonal interactions can be affected by this new routine of wearing surgical masks when we are in public places. An important aspect of successful interactions in a social context is remembering to whom we relay information, a type of memory defined as destination memory (Gopie and MacLeod 2009).

Destination memory can be considered episodic memory since it involves the reconstruction of the context in which an event has occurred. It is recollected in the context of a particular time and place concerning oneself as a participant in the episode (Gopie and MacLeod 2009). Although this type of memory has only recently been studied, research suggests that destination memory is associated with greater communication effectiveness and better interaction with others (Gopie and MacLeod 2009; Johnson and Jefferson 2018).

In destination memory studies, familiar faces (e.g., celebrity faces) are often used because, in everyday life, we mainly transmit information to people we know (i.e., family, friends, and/or colleagues). Indeed, in a study that compared destination memory between younger and older adults, better destination memory for familiar faces (i.e., celebrity faces) than unfamiliar ones was found, suggesting that previous knowledge about the person to who we share information leads to better destination memory (El Haj et al. 2015).

Besides recipients' face familiarity, other variables related to destination memory have been studied. For instance, Gopie and MacLeod (2009) studied the importance of the attentional focus on the recipient by asking participants to say the recipient’s name before sharing the information. It seems that when the name is spoken, there is an increase in the attentional focus on the context (i.e., the destination person’s face), promoting the association between the face and the fact and, consequently, improving destination memory. In another study, Barros et al. (2021) tested the importance of variables associated with the recipient’s face: by presenting faces with distinctive features (e.g., a tattoo or a scar) and faces without unique features, destination memory performance was better when participants shared the information with faces with distinctive features (Barros et al. 2021). The authors assumed that when the face displayed different features, it was perceived as different from others (i.e., a more distinctive face), attracting greater attention during encoding and producing a stronger association between the recipient's face and the proverb (Barros et al. 2021).

In sum, the previous studies (Barros et al. 2021; Gopie and MacLeod 2009) have observed better destination memory performance when participants shift the attentional focus from themselves and from the processes of sharing the information to the person to whom that information is being transmitted. This change leads the participant to pay more attention to the face–proverb pair, thus increasing the associative link between the face and the proverb.

Considering that variables associated with the recipient’s face can lead to changes in destination memory performance, it seems likely that presenting faces using a surgical mask, a variable that has been established as disrupting face recognition (Carragher and Hancock 2020; Freud et al. 2020), could hinder destination memory as well. However, it is still unclear how wearing face masks can ultimately affect destination memory. Our experiment aimed to understand the effect of wearing surgical masks on destination memory. We expected participants to have lower destination memory performance when sharing information with masked celebrities than when sharing information with an unmasked face (Carragher and Hancock 2020; Freud et al. 2020).

## Experiment 1

### Method

#### Participants

Experiment 1 sample consisted of 40 undergraduate students (34 females) between 18 and 26 (*M*_age_ = 19.75, SD = 1.30). The sample size was calculated a priori through G*Power (Faul et al. 2007), using an alpha (*α*) of 0.05, a large effect size (Cohen's *d* = 0.80), and a statistical power of 0.80. A large effect size was chosen taking into account the literature in the area of destination memory, more specifically, one of the pioneering studies in this area, whose procedure was replicated in this study (experiment 1: *η*2 = 0.90; Gopie and MacLeod 2009). Participants were native European Portuguese speakers and had a normal or corrected-to-normal vision. Written consent was obtained from all participants who received course credits for their participation. The local ethics committee approved this study.

#### Materials

##### Proverbs

Sixty Portuguese proverbs with 4 to 10 words (e.g., "*A pressa é inimiga da perfeição*"^1^) were selected from a previous study (Barros et al. 2021), where the proverbs were evaluated using a five-point scale regarding familiarity level (0 = very unfamiliar proverb; 5 = very familiar proverb) and emotional valence (0 = very negative proverb; 5 = very positive proverb). The choice of proverbs instead of another type of information was based on the premise that when information is familiar, more cognitive resources are available to memorize the association between the information and the face (El Haj et al. 2015). For this reason, proverbs were selected to have an average familiarity of above 4 on a 5-point Likert scale that varied between 1 and 5 (1 = very unfamiliar proverb; 2 = unfamiliar proverb; 3 = neutral proverb; 4 = familiar proverb; 5 = very familiar proverb. Additionally, to ensure that proverbs’ emotionality did not influence the results, we selected proverbs with neutral emotional valence, with values between 2.25 and 3.75 on a Likert scale that ranged from 1 to 5 (1 = very negative proverb; 2 = negative proverb; 3 = neutral proverb; 4 = positive proverb; 5 = very positive proverb). The proverbs were presented in white font against a black background.

##### Faces

Sixty celebrity faces (e.g., Barack Obama) were selected from a celebrity database validated for the Portuguese population using the same age group—young adults (Lima et al. 2021). In this database, 160 celebrity faces pictures were evaluated for several measures, like naming and recognition rates, presenting male and female celebrity faces and national and international celebrity faces (Lima et al. 2021). Celebrity faces were used because, in everyday life, we mainly transmit information to recipients who are known or familiar to us and because we could more easily infer that they were familiar to our sample (Lima et al. 2021). Considering this argument, we selected faces with naming accuracy above 75% (*M* = 91.63; SD = 5.33) and recognition accuracy above 80% (*M* = 97.17; SD = 3.61). The images selected were also controlled regarding background (i.e., Portuguese and international celebrities) and gender (i.e., male and female), with all of these variables being presented in equal proportions.

**Fig. 1:**
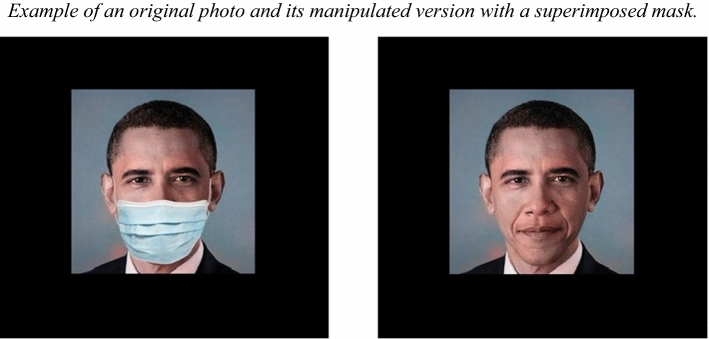
Example of an original photograph and its manipulated version with a superimposed mask

Every celebrity image was duplicated, with one of the images being manipulated with adobe photoshop CC (Adobe Systems Incorporated 2014): we superimposed a standard surgical mask on the original photograph (see Fig. 1), a technique usually used in other studies (e.g., Carragher and Hancock 2020). In Experiment 1, each of the 60 faces had two versions: unmasked (original photograph) and masked face (manipulated photograph). The faces were presented in color against a black background, and all stimuli were presented at the center of the computer screen.

#### Design

The independent variables were the presence of masks (masked faces vs. unmasked faces) and the type of stimuli (proverbs vs. faces). The first variable was manipulated through a between-participants design, where half of the participants transmitted information to masked faces, and the other half transmitted information to unmasked faces. The type of stimuli was manipulated through a within-participants design. This design allowed us to replicate the paradigm Gopie and MacLeod (2009) employed, applying the same type of design and the same number of stimuli.

To understand whether the use of masks influenced item memory (i.e., memory for the proverbs and memory for the face presented) and destination memory (e.g., memory for the association between the proverbs told and the faces they were told to), sensitivity index or d′ score [z(P(hits) − z(P(false alarms))] and response bias (c) score [−  (z (hits) + z (false alarms))/2] were calculated for all recognition memory tests (face memory test, proverb memory test, and destination memory test) on each independent variable condition. Hits refer to “yes” responses to the face–proverb pairs, proverbs, and faces that were presented in the study phase (correct “yes” response), and false alarms refer to “yes” responses to face–proverb pairs, proverbs, and faces that were not presented in the study phase (incorrect “yes” responses).

#### Analysis

A higher d´ score means that participants were better at identifying faces and proverbs presented in the study phase (in the item memory task) and at identifying to which celebrity they transmitted the proverb (in the destination memory task). In other words, these participants had a higher number of hits and a lower number of false alarms. Regarding the response bias (c) score, a higher c score means that participants were more conservative in their responses (more “no” responses). In comparison, a lower c score means that participants were more liberal (more “yes” responses).

To observe the impact of masks on destination memory, an independent samples t-test to destination memory performance (i.e., *d*´ values on destination memory task) was conducted, in which we compare the transmission of proverbs to masked faces to the transmission of proverbs to unmasked faces.

To observe whether the presence of a mask and the type of stimuli influenced item memory, we also applied to *d*′ values a 2 (mask presence: masked faces vs. unmasked faces) × 2 (type of stimuli: faces vs. proverbs) mixed ANOVA, with the first independent variable manipulated between-participants and the other within-participants.

#### Procedure

The study was conducted remotely: the participant would enter a video call with the researcher using the software Zoom (Zoom Video Communications, Inc. 2020). Then, the researcher shared their screen, where stimuli presentation and response recording were controlled with E-Prime 3.0 (Psychology Software Tools, Inc. 2016). So, the procedure ran on the researcher’s computer, and the remote control was given to participants so they could answer the procedure. First, informed consent was obtained from participants, and a sociodemographic questionnaire was completed.

**Fig. 2:**
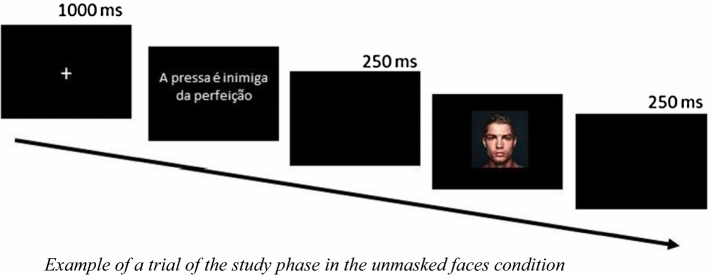
Example of a trial of the study phase in the unmasked faces condition

The main procedure included two phases: the study and the test phases. In the study phase, participants told 50 proverbs aloud to 50 celebrity faces: the 50 proverbs were randomly paired with the 50 faces. They were not told that their memory would be tested later. Each study trial started with a white fixation cross on a black background for 1000 ms, and then, a proverb was presented. After reading silently and memorizing the proverb, participants were instructed to press the keyboard spacebar. This resulted in a blank screen presented for 250 ms, followed by a color picture of a celebrity face. Here, participants had to say aloud to the face the proverb they had just read and then press the spacebar again, resulting in another blank screen for 250 ms. This procedure was repeated until the participant had told all the 50 proverbs to the 50 faces (see Fig. 2). Participants were randomly assigned to one of the conditions: faces with masks or faces without masks. It is important to note that in the masked faces condition, all celebrities were presented with surgical masks, both in the study and test phases. On the other hand, in the unmasked faces condition, all the faces were presented without masks in both phases.

After the study phase, all participants completed two recognition memory tests presented in a counterbalanced order: the item and the associative memory tests. These two tests used entirely non-overlapping sets of stimuli to prevent cross-test contamination. Forty items (20 proverbs and 20 faces) were presented randomly and individually on the item memory test. Half of the items were stimuli previously presented in the study phase (i.e., targets), and the other half were not (i.e., distractors). Each face or proverb remained visible until the participant indicated whether that item had appeared during the study phase. In other words, the participants had to answer the following questions: "Did you say this proverb in the previous phase?" or "Did you see this person in the previous phase?" The participant responded ‘‘yes’’ by pressing the ‘‘c’’ key on the computer keyboard and ‘‘no’’ by pressing the ‘‘m’’ key. Once a response was made, a blank screen was displayed for 250 ms, and the subsequent test trial followed.

On the associative memory test, 40 face–proverb pairs were shown in random order: twenty pairs had been presented during the study phase, and the other 20 were random re-pairings of previously studied proverbs and faces. Face–proverb pairs were presented one at a time, with the proverb below the face. Participants reported whether they had previously told that proverb to that face. “Yes’’ and ‘‘no’’ responses were made by pressing the same keys as in the item memory test. Each pair remained visible until the participant responded. After each response, a blank screen was displayed for 250 ms, and then, the subsequent test trial was presented. The entire procedure took approximately 20–30 min.

### Results

The mean proportions of hits, false alarms, and *d′* values are shown in Table 1. The software used for the data analysis was JASP 0.11.1 (Team JASP 2020).

**Table 1:** Mean proportion of hits and false alarms to item memory and destination memory, and *d′* values as a function of condition

	Hits	False alarms	*d*′
*Masked faces condition*			
Item memory: proverbs	0.81 (0.15)	0.03 (0.06)	2.55 (0.57)
Item memory: faces	0.81 (0.17)	0.19 (0.15)	1.93 (0.60)
Destination memory	0.63 (0.15)	0.39 (0.12)	0.64 (0.58)
*Unmasked faces condition*			
Item memory: proverbs	0.86 (0.17)	0.03 (0.04)	2.73 (0.56)
Item memory: faces	0.85 (0.15)	0.07 (0.08)	2.48 (0.57)
Destination memory	0.69 (0.12)	0.32 (0.15)	1.07 (0.59)

Standard deviation of the mean is reported in parenthesis

#### Destination memory

We applied an independent samples *t*-test to destination memory performance. Concerning *d′*, results showed better memory for unmasked faces in the destination memory, *t*(38) = 2.34, *p* = 0.03, Cohen’s* d* = 0.74, 95% CI [0.09, 1.38]. The *t*-test applied to the response bias (c) showed no statistically significant difference between the conditions *(p* > 0.05).

#### Item memory

We also applied a 2 (mask presence: masked faces vs. unmasked faces) × 2 (type of stimuli: faces vs. proverbs) mixed ANOVA to item memory *d′* values. Results showed a main effect of type of stimuli with proverbs being better recognized than faces, *F*(1, 38) = 13.03, *p* < 0.001, *η*_*p*_^*2*^ = 0.12; and, also the main effect of mask presence, *F*(1, 38) = 2.73, *p* = 0.01, *η*_*p*_^*2*^ = 0.09, with better item memory on the unmasked faces condition. The interaction between the variables was not significant, *F*(1, 38) = 2.45, *p* = 0.13, *η*_*p*_^*2*^ = 0.06.

Finally, using c, we ran a mixed 2$\times$2 ANOVA considering the same variables. Results showed a main effect of type of stimuli with proverbs having a more conservative criterion than faces, *F*(1, 38) = 7.97, *p* = 0.01, *η*_*p*_^*2*=^0.17. The main effect of mask presence was not significant, *F*(1, 38) = 0.16, *p* = 0.69, *η*_*p*_^*2*=^0.004, nor was the interaction, *F*(1, 38) = 3.83, *p* = 0.06, *η*_*p*_^*2*^ = 0.09.

### Discussion

Considering that variables associated with the recipient’s facial features can lead to changes in destination memory (Barros et al. 2021) and that during interpersonal interactions, people spend a considerable amount of time looking at the other’s face (Broz et al. 2012; Rogers et al. 2018), the present work aimed to understand whether using surgical face masks would impair the ability to remember to whom we relay information. Destination memory is a recent area of research, and although not extensively studied, we must understand this type of memory since when we are not capable of remembering to whom we told something, we tend to repeat the same information to the same person. This is one of the consequences of a faulty destination memory, redundancy, which consists of repeating the same story multiple times to the same recipient (Kausler and Hakami 1983).

Evidence showed that different facial features could contribute differently to face recognition; namely, the importance of upper facial features for familiar faces was previously observed (Sadr et al. 2003). However, despite the importance of upper facial features on face recognition, results have shown that wearing face masks may affect face processing and, consequently, facial recognition (Carragher and Hancock 2020; Freud et al. 2020). Nevertheless, no study has noted the influence of masks on destination memory. Considering that variables associated with the recipient’s face can lead to changes in destination memory performance, it seems likely that presenting faces using a surgical mask could also hamper destination memory. For this reason, our experiment aimed to understand whether presenting a destination memory procedure using masked celebrities would hinder destination memory.

As expected, our results showed that participants had worse destination memory performance when sharing information with masked celebrities than unmasked celebrities. Also, we observed lower recognition of masked faces (*M* = 1.93) than unmasked faces (*M* = 2.49), which supports the finding that the use of surgical face masks makes it harder to recognize a face (Carragher and Hancock 2020; Freud et al. 2020). These results were expected since using a surgical mask produces a disruption in face processing, affecting facial recognition (Carragher and Hancock 2020; Carbon 2020; Freud et al. 2020), consequently making it harder to recognize whether it was to the face presented to the participant that a previous transmission of specific information has occurred. Additionally, our results showed a main effect of type of stimuli, with proverbs being better recognized than faces.

We can conclude that our results are in line with previous studies that have shown that wearing face masks may affect facial recognition (Carragher and Hancock 2020; Freud et al. 2020) and with prior studies demonstrating that variables associated with the recipient’s face could lead to changes in destination memory performance (Barros et al. 2021; Gopie and MacLeod 2009).

However, since experiment 1 was conducted using a between-participants design, it is not possible to clearly say that surgical masks produced this effect or if it is only a group effect. Considering this, we conducted a second experiment, following the Experiment 1 procedure but with a within-participants design.

## Experiment 2

Most studies in the field of destination memory applied a between-participants design since the goal was to compare the destination memory performance of normative population with populations with several disorders (e.g., Alzheimer's disease: El Haj et al. 2013; Schizophrenia: El Haj et al. 2017; Korsakoff's syndrome: El Haj et al. 2016b; Huntington's disease: El Haj et al. 2016a, b) and between young and older samples (El Haj et al. 2013; Gopie et al. 2010). Few studies applied a within-participants design (Barros et al. 2021; El Haj et al. 2018; El Haj and Ndobo 2021).

However, to observe how the presence of a mask interferes with the memory mechanisms involved in face recognition, Garcia-Marques et al. (2022) performed three experiments applying different designs (Experiment 1: between-participants design; Experiment 2 and 3: within-participants design). The authors suggest that the use of a within-participants design allows inferring about the interference of different types of materials (in this case, faces with and without masks) exert over each other. It is particularly important since, in a real-life context, this interference is likely to occur naturally. So, although most destination memory studies apply between-participants designs, in experiment 2, our goal was to observe if the same pattern of results was obtained when implementing a within-participants design.

### Method

#### Participants

Experiment 2 sample consisted of 45 undergraduate students (36 females) between 18 and 28 (*M*_age_ = 21.98, *SD* = 3.09). The sample size was calculated a priori through G*Power (Faul et al. 2007), using an alpha (*α*) of 0.05, a medium effect size (*Cohen's d* = 0.50), and a statistical power of 0.95. A medium effect size was chosen taking into account the study by Barros et al. (2021, Exp. 1), where a variable associated with the recipient’s face (i.e., face distinctiveness) was manipulated, and a within-participants design was applied.

Participants were native European Portuguese natives and had a normal or corrected-to-normal vision. Participants that took part in Experiment 1 could not take part in experiment 2. Written consent was obtained from all participants who received course credits for their participation. The local ethics committee approved this study.

#### Materials

Materials were the same as those used in experiment 1. However, we added four more proverbs and celebrity faces to ensure that the participant saw the same number of masked female faces, masked male faces, unmasked female faces, and unmasked male faces. For the additional faces and proverbs, we maintained the same criteria: proverbs were selected to have an average familiarity above 4 and a neutral emotional valence, with values between 2.25 and 3.75 on a Likert scale that varied between 1 and 5; and the selected faces had above 75% in naming accuracy and 80% in recognition accuracy. Also, it is important to note that the stimuli were counterbalanced so that each celebrity face was presented with a mask for some participants and without a mask for the rest.

#### Design

The independent variables were the presence of masks (masked faces vs. unmasked faces) and the type of stimuli (proverbs vs. faces). In this experiment, both variables were manipulated through a within-participants design, where participants transmitted information to masked and unmasked faces. We measured the same dependent variables, the sensitivity index or *d′* score and the response bias (c) score.

#### Analysis

To observe the impact of masks on destination memory, a paired samples t-test to destination memory performance (i.e., *d*′ values on destination memory task) is used, in which we compare the transmission of proverbs to masked faces with the transmission of proverbs to unmasked faces.

To observe whether the presence of a mask and the type of stimuli influenced item memory, we applied a 2 (mask presence: masked faces vs. unmasked faces) × 2 (type of stimuli: faces vs. proverbs) repeated measures ANOVA, with the two independent variables manipulated in a between-participants design.

#### Procedure

Experiment 2 followed the same procedure as experiment 1 with one exception: all participants transmitted information to both masked and unmasked faces. This experiment was conducted online.

The main procedure included the same two phases: the study and the test phase. In the study phase, participants told aloud 52 proverbs to 26 masked celebrity faces and 26 unmasked celebrity faces. Participants were not told that their memory would be tested later. It is important to note that if a face was presented with a mask in the study face, it would be presented with a mask in the test phase. And a face presented without a mask in the study phase would be presented without a mask in the test phase.

Like in experiment 1, all participants completed two recognition memory tests presented in a counterbalanced order: the item and the associative memory tests. The entire procedure took approximately 20–30 min.

### Results

The mean proportions of hits, false alarms, and *d′* values are shown in Table 2. The software used for the data analysis was JASP 0.11.1 (Team JASP 2020).

**Table 2:** Mean proportion of hits and false alarms to item memory and destination memory, and d′ values as a function of condition

	Hits	False Alarms	d′
*Masked faces condition*			
Item memory: proverbs	0.77 (0.23)	0.02 (0.07)	2.17 (0.77)
Item memory: faces	0.91 (0.14)	0.16 (0.21)	2.19 (0.82)
Destination memory	0.70 (0.18)	0.35 (0.16)	1.05 (0.71)
*Unmasked faces condition*			
Item memory: proverbs	0.79 (0.16)	0.07 (0.11)	2.07 (0.56)
Item memory: faces	0.91 (0.13)	0.09 (0.12)	2.38 (0.55)
Destination memory	0.73 (0.16)	0.28 (0.17)	1.32 (0.77)

Standard deviation of the mean is reported in parenthesis

#### Destination memory

We applied a paired samples *t*-test to destination memory *d′* results. The analysis revealed that destination memory was worse when participants shared information with masked faces (*M* = 0.87, *SD* = 0.74) compared with unmasked faces (*M* = 1.15, *SD* = 0.74), *t*(44) = 2.41, *p* = 0.02, Cohen's *d* = 0.36, 95% CI [0.06, 0.66]. Finally, the *t*-test applied to the response bias (c) showed no statistically significant difference between conditions *(p* > 0.05).

#### Item memory

Also, we performed a 2 (Mask presence: masked faces vs. unmasked faces) × 2 (Type of stimuli: proverbs vs. faces) repeated measures ANOVA on the item memory *d′* data. There was no main effect of mask presence, *F*(1, 44) = 1.34, *p* = 0.29, *η*_*p*_^*2*^ = 0.03, and there was no main effect of type of stimuli, *F*(1, 44) = 0.02, *p* = 0.88, *η*_*p*_^*2*^ = 0.01. However, there was an interaction between the mask presence and the type of stimuli, *F*(1, 44) = 10.01, *p* = 0.003, *η*_*p*_^*2*^ = 0.19, where unmasked faces (*M* = 2.34) were better recognized in the item memory test than masked faces (*M* = 2.01).

Finally, using c criteria, we ran a mixed 2 × 2 ANOVA considering the same variables. Results showed a main effect of type of stimuli with proverbs having a more conservative criterion than faces, *F*(1, 44) = 30.02, *p* < 0.001, *η*_*p*_^*2*^ = 0.41. The main effect of mask presence was not significant (*p* = 0.69), nor was the interaction (*p* = 0.06).

### Discussion

Some differences regarding the item memory performance between experiments were found. In experiment 1, we observed better item memory for proverbs than faces, a result that is not replicated in this second experiment (i.e., we did not observe a main effect of type of stimuli in item memory). However, in both experiments, participants were more conservative (i.e., they gave more “no” responses”) for proverbs than for faces. Interestingly, these experiments allowed us to conclude that the response criterion is not directly related to memory performance. Participants were more conservative in their responses for proverbs than for faces in both experiments, but only in experiment 1 participants had better item memory for facts than for faces.

Also, in the second experiment, we did not observe better item memory in the unmasked faces condition as in experiment 1 (i.e., we did not observe a main effect of mask presence in item memory). This can suggest that item memory performance is more dependent on the type of design implemented. It is important to note that in experiment 1, participants saw all faces with a mask or all faces without a mask. In experiment 2, participants saw half of the faces with masks and the rest without.

However, and more important for the purpose of our study, as observed in Experiment 1, our results showed that participants had worse destination memory performance when sharing information with masked celebrities than unmasked celebrities. Congruently with experiment 1, we also observed lower recognition of masked faces than unmasked faces, which supports the finding that surgical face mask use makes it harder to recognize a face (Carragher and Hancock 2020; Freud et al. 2020).

These results provide robustness to experiment 1 results and to previous studies that showed that wearing face masks may affect facial recognition (Carragher and Hancock 2020; Freud et al. 2020). With these two experiments, we can conclude that the use of masks, besides affecting the memory for faces, also affects the ability to remember to whom we relay information (i.e., destination memory).

## General discussion

As referred before, destination memory involves remembering to whom we told something. Although this type of memory has been studied only recently (Gopie and MacLeod 2009), research suggests that destination memory is associated with greater communication effectiveness and better interaction with others (Gopie and MacLeod 2009; Johnson and Jefferson 2018). Besides allowing us to maintain successful interpersonal interactions, destination memory is also essential in the workplace, for example, when supervisors need to remember to whom they delegated a specific task. This is why it is important to understand which variables could potentially decrease or improve destination memory performance.

Several studies have shown the importance of the attentional focus on the information’s recipient and of the variables associated with its face for better destination memory performance (Barros et al. 2021; Gopie and MacLeod 2009). Also, several studies have shown the impact of masks on facial recognition (Carragher and Hancock 2020; Freud et al. 2020). However, no study had been conducted to observe the impact of masks on other types of memory, namely destination memory. So, our experiment aimed to understand the effect of wearing surgical masks on destination memory. We hypothesized that participants would have lower destination memory when sharing information with masked celebrities than with unmasked celebrities (Carragher and Hancock 2020; Freud et al. 2020).

Indeed, in both experiments, our results showed that participants had worse destination memory performance when sharing information with masked celebrities than unmasked celebrities. Also, we observed lower recognition of masked faces than unmasked faces. These results align with previous studies that show that wearing face masks may affect facial recognition (Carragher and Hancock 2020; Freud et al. 2020). More importantly, these results also support the idea that variables associated with the recipient’s face are critical for understanding destination memory and, therefore, must always be considered.

One possible explanation for worse destination memory performance in the presence of masks can be face distinctiveness. Previously, better destination memory performance was observed when participants shared information with people whose faces had distinctive features (e.g., tattoos, piercings, color hair, etc.) than faces that displayed no distinctive features (Barros et al. 2021). A higher distinctiveness of the recipients’ faces will make these faces stand out and be perceived as different from the others, attracting more attention to themselves during the encoding. As previously observed, when the participant shifts the focus of attention from himself and from the processes of sharing that information to the person to whom that information is being transmitted, there is better destination memory performance (Barros et al. 2021; Gopie and MacLeod 2009).

However, better destination memory for faces with distinctive features was only observed in a within-participants design (Barros et al. 2021). The authors explained it with the necessity of comparing stimuli (in their case, between distinctive and non-distinctive features) for a distinctiveness effect to be found. In our study, the effect of the mask on destination memory was observed in both designs (experiment 1 and experiment 2). Although, when we presented faces with masks, besides resulting in a lower facial distinctiveness, face processing was disrupted. So, our results can be explained based on these two ideas: masked faces are less distinctive, and their holistic processing is disrupted.

In future studies, and to understand the influence of surgical masks on facial distinctiveness, the presence of distinctive features (faces with and without distinctive features) and the mask presence (faces with and without masks) should be manipulated. This study would allow us to observe whether distinctive features mitigate the impact of masks on facial recognition and destination memory. Although previous studies have shown that upper facial features are important for face recognition, particularly of familiar faces, observing the upper face was insufficient to mitigate the influence of masks on facial recognition and destination memory performance. For this reason, it might be interesting to see if the distinctive features that lead to better facial recognition and destination memory can or not mitigate the influence of mask use.

It is also important to note that our studies were conducted with a young, Western sample; therefore, these results may not be generalized to other cultures or religions. In future studies, it would be interesting to observe if the same pattern of results on the item and destination memory tasks are observed in contexts in which face occlusions are typical in everyday life, like religious contexts. For example, it was previously observed that a vast experience with faces framed by a headscarf might train the ability to accurately process internal face information, in which Emirati participants had better face recognition than American participants (Wang et al. 2015). Probably, the impact of mask use may not be so significant on the item and destination memory tasks with Emirati participants since, in everyday life, it is more common for them to observe and transmit information to covered faces.

In sum, we can conclude that participants had worse destination memory performance when sharing information with masked celebrities than unmasked celebrities. Also, we observed worst recognition of masked faces when compared to unmasked ones. These results were expected since using a surgical mask affects facial recognition (Carragher and Hancock 2020; Freud et al. 2020), thus making it harder to recognize a person to who the information was previously transmitted. More importantly, these results support the idea that variables associated with the recipient’s face are important for destination memory performance (Barros et al. 2021; Gopie and MacLeod 2009).

## Footnotes

1 An approximate English translation of the Portuguese proverb is “*Haste is the enemy of perfection”.*
